# Fire-scarred fossil tree from the Late Triassic shows a pre-fire drought signal

**DOI:** 10.1038/s41598-020-77018-w

**Published:** 2020-11-18

**Authors:** Bruce A. Byers, Lucía DeSoto, Dan Chaney, Sidney R. Ash, Anya B. Byers, Jonathan B. Byers, Markus Stoffel

**Affiliations:** 1 Bruce Byers Consulting, Falls Church, VA USA; 2 MedDendro Lab, Centre for Functional Ecology, University of Coimbra, Coimbra, Portugal; 3 Experimental Station of Arid Zones, Spanish National Research Council (CSIC), Madrid, Spain; 4 National Museum of Natural History, Washington, DC USA; 5 University of New Mexico, Albuquerque, NM USA; 6 The Nature Conservancy, Boulder, CO USA; 7 Department of Geography, University of Montana, Missoula, MT USA; 8 Climate Change Impacts and Risks in the Anthropocene, Institute for Environmental Sciences, University of Geneva, Geneva, Switzerland; 9 dendrolab.Ch, Department of Earth Sciences, University of Geneva, Geneva, Switzerland; 10 Department F.-A. Forel for Aquatic and Environmental Sciences, University of Geneva, Geneva, Switzerland

**Keywords:** Palaeoecology, Palaeoclimate

## Abstract

Exploring features of wood anatomy associated with fire scars found on fossil tree trunks is likely to increase our knowledge of the environmental and ecological processes that occurred in ancient forests and of the role of fire as an evolutionary force. In Petrified Forest National Park, Arizona, where Late Triassic fossil trees are exposed, we found 13 examples of fossil logs with external features resembling modern fire scars. One specimen with the unambiguous external features of a fire scar was collected for analysis of its fossilized wood. A light-colored band composed of compressed and distorted tracheids was associated with the scarring event. Cell lumen diameter and cell wall thickness in the pre-scarring fossilized wood show a response similar to that described in modern trees experiencing drought conditions. Tracheids in the post-scarring wood are initially smaller, and then become larger than average following a recovery period, as is often observed in modern conifers following fire. The responses in external morphology and wood anatomy to drought and fire were similar to those of some modern trees and support the view that some forests may have experienced conditions favoring the evolution of fire-adapted traits for more than 200 million years.

## Introduction

Fossil charcoal (fusain) in the geological record provides evidence that forest fires are an ancient ecological phenomenon, going back to at least the Devonian Period at around 400 Ma (ref.^1^). Fossil charcoal found in the Upper Triassic Chinle Formation in Petrified Forest National Park has been taken as evidence of ancient wildfires^2^. Charcoal has also been reported from Late Triassic sandstones in southwestern Germany^3^. Polycyclic aromatic hydrocarbons (PAHs) created by pyrolysis of organic matter and deposited in sediments provide geochemical evidence for ancient wildfires^4^. Studies of charcoal and pyrolytic PAHs in sediments suggest increasing wildfire activity through the Triassic and across the Triassic-Jurassic boundary in many places, such as Poland, Denmark, Greenland, and the Chinle Basin^4–7^.

The apparent increase in paleowildfire activity through the Late Triassic has led to speculation about its causes. Recent investigations using paleosol geochemistry indicate that the Chinle Basin was becoming increasingly arid in the Late Triassic^8,9^. New quantitative palynological data from the Chinle Formation show that a floral turnover occurred between 217 and 213 Ma, reflecting a gradual transition toward a more arid climate^10^. A recent study that included proxy information about both climate and wildfire in the Late Triassic Chinle Basin concluded that the climate was generally arid and strongly fluctuating, with elevated and increasing atmospheric CO_2_ levels, and that wildfires were common and widespread^7^. Based on a large increase in fossil charcoal observed in Greenland across the Triassic-Jurassic boundary, it has been speculated that climate warming and high CO_2_ may have led to a shift in species composition of plant communities (then composed mainly of ginkgoes, conifers, and ferns/cycads) from predominantly less flammable broad-leaved species to more flammable narrow-leaved species, increasing the fire activity^11^.

Although the presence of charcoal and pyrolytic PAHs in the paleoenvironmental record provide evidence that fires burned in ancient forests, it does not provide direct evidence about ancient fire regimes in terms of frequency, magnitude (severity and intensity), size, seasonality, and spatial patterns^12,13^. Fire-adapted traits confer a selective advantage and increase plant fitness in fire-prone environments^14,15^, and different suites of fire-adapted traits have evolved under different fire regimes. For example, serotiny (i.e., the retention of seeds in closed cones or woody fruits until synchronous release is triggered by fire) is commonly observed in present-day northern hemisphere conifer forests with high-intensity crown-fire regimes, while thick bark and self-pruning growth forms are adaptive in low-intensity surface-fire regimes^16^.

Analyses of fire scars in modern forests provide the information needed to develop an understanding of fire regimes^12,13,17,18^. Fire scars can be identified by a diagnostic suite of external and internal features not found together in any other type of tree scar. The characteristic external morphology of fire scars is the result of the flame dynamics of passing surface fires that wound the tree, but do not kill it^19^. Fire-scarred trees also exhibit distinctive patterns of internal wood anatomy. A band of compressed and distorted tracheids is typically formed as an immediate response to wounding by fire, often creating a visible scar-associated band at the same radius as the fire event, as has been described in both modern trees^20^ and in a fire-scarred fossil tree^21^. Following this immediate wounding response, tracheids were found to be much smaller during a post-fire recovery period in three North American conifers^22,23^.

Fire-scarred modern conifers often exhibit an increase in ring widths, known as “growth release,” after they have recovered from the fire^24–28^. Growth releases are considered a reliable “fire indicator”^24^ and are generally attributed to reduced competition for light, nutrients, and/or water caused by the mortality of nearby trees or other vegetation, and/or to an increase in available nutrients released from burned vegetation or litter^26,28^. In gymnosperms, where wood is mainly composed of tracheids, the formation of wide rings following fires results from an increase in tracheid size and/or tracheid number^22,29^.

Past work on modern vegetation has found associations between droughts and forest fires^24,30–34^. Tree-ring width has been used as a proxy for drought in conifer species, including *Araucaria araucana*^30,31,34^ and *Sequoiadendron giganteum*^24^, thereby allowing reconstructions of climate history beyond systematic records. Trees are known to respond physiologically to water stress by altering their tracheid size and number^35–37^. Cavitation resistance is a critical determinant of drought tolerance in trees, and smaller, thicker-walled tracheids provide better resistance against loss of hydrological conductivity caused by water stress^38,39^.

We analyzed the microscopic wood anatomy of a fossil tree trunk with the characteristic external features of a fire scar from Petrified Forest National Park, Arizona, USA. The aim was to identify and interpret environmental conditions prevailing immediately before the paleowildfire. In particular, we wanted to understand whether drought conditions preceded the fire, as is often the case in modern forests, and whether the tree responded to wounding by fire as modern trees do. We hypothesized that the fossil wood anatomy would show (1) a signal of pre-scarring water stress from a physiological response to drought immediately before the scarring event; (2) a scar-associated band of compressed and distorted tracheids; (3) an immediate post-scarring decrease in tracheid size during a recovery period; and (4) an increase in tracheid size after post-fire recovery. We discuss the relevance of our results to understanding the evolution of fire-adapted plant traits.

## Results

### Geological setting

**Figure 1:**
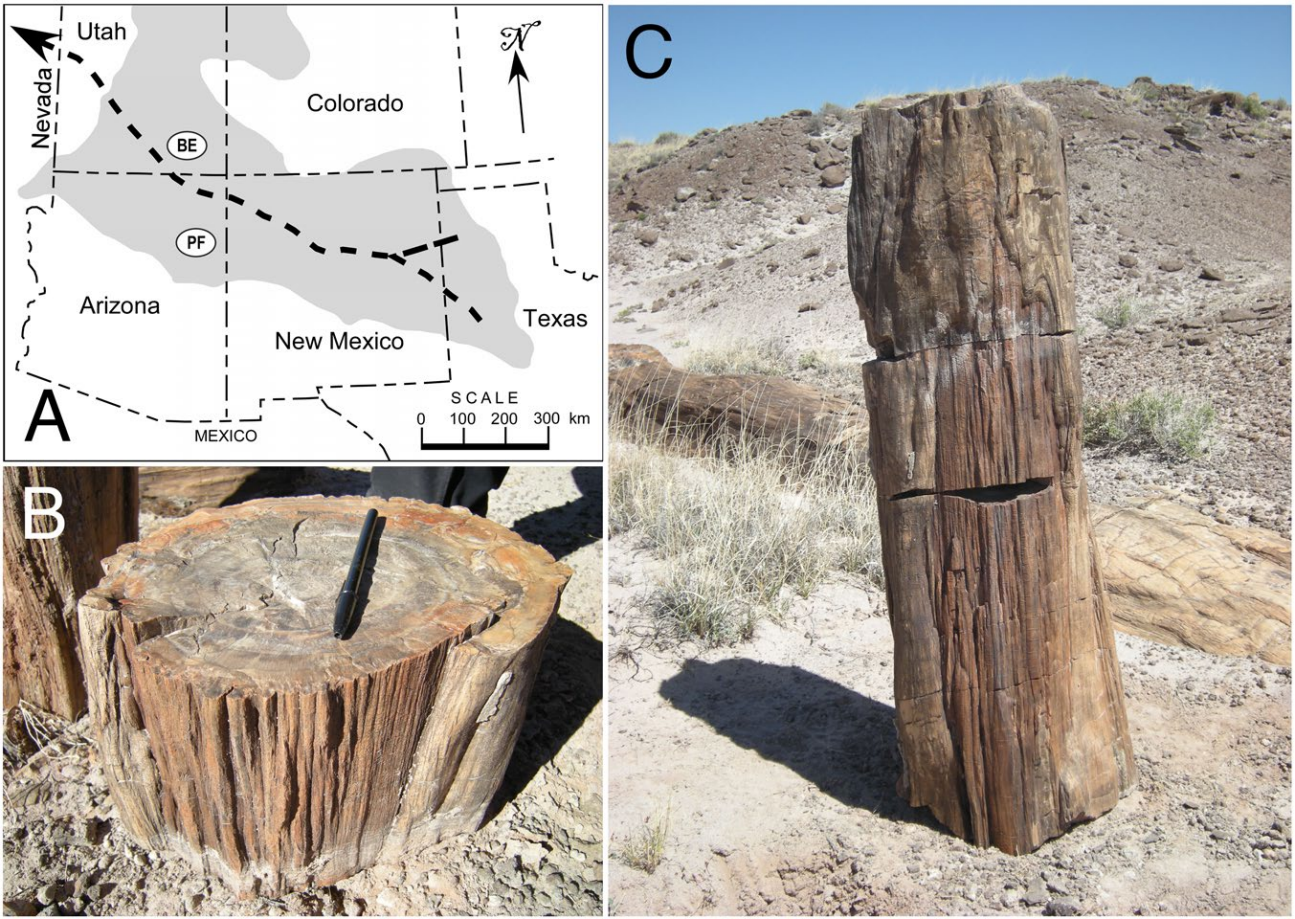
Location and in situ specimen. (**A**) Current location of the Chinle Formation (grey shading), the course of the Chinle-Dockum paleo-river system that flowed from what is now northwestern Texas to Nevada (heavy dashed lines), and locations of Bears Ears (BE) and Petrified Forest (PF). (**B**) Trunk section that was removed and analyzed; in this photo the section is inverted, with lower side up (15 cm pen for scale). (**C**) Upper three sections above root base with fire scar features; specimen analyzed is middle section.

The section of fossil tree trunk analyzed here was found during a search for potential fossil fire scars conducted in the Petrified Forest National Park in October 2013. The Petrified Forest is about 300 km south of Bears Ears, Utah, where the first example of a fossil fire scar described in the literature was found^21^. The specimen described here was found in the Black Forest Bed, which occurs at the top of the Petrified Forest Member of the Chinle Formation of Late Triassic age (Norian-Rhaetian Stages) in the Painted Desert area of the national park^40^. The Chinle Formation was deposited by meandering rivers and streams in an environment of lakes, ponds, and marshes between about 225 and 203 Ma (ref.^41^). The Black Forest Bed has been dated to about 210 Ma^42,43^. The east–west flowing Chinle-Dockum paleo-river system crossed a broad continental back-arc basin that was originally located about 5–15° N of the paleo-equator near the western edge of Pangea^44,45^. This fluvial system originated on the craton in what is now west Texas and flowed westward across northern New Mexico and Arizona into southwestern Utah, before eventually emptying into the sea in southeastern Nevada^46^ (Fig. 1A). The paleo-river system existed in some form until about 208 Ma, after which the basin dried out except for a small area in northeastern Arizona. This aridification of the Petrified Forest paleolandscape was due to a rain shadow developing on the leeward side of the rising Cordilleran magmatic arc in California, about 400 km west of the Chinle-Dockum basin^44^ . Climate at the time of the Black Forest Bed deposition was sub-humid and warm temperate, but becoming increasingly hotter and drier^8^.

The Black Forest Bed contains fossil logs and in situ stumps of *Agathoxylon arizonicum*, *Pullisilvaxylon arizonicum,* *P. daughertii, Schilderia adamanica*, and *Woodworthia arizonica*, and fragments of *A. arizonicum* wood that have been abraded during water transport^47,48^. The logs and stumps in the Black Forest Bed range up to nearly a meter in diameter and some are more than 50 m long. Because fire scars are found at the base of trees, the fact that in situ stumps exist in this bed makes it a good place to look for fossil fire scars.

### Description of specimen and macroscopic features of the scar

The section of fossilized *A. arizonicum* trunk that was collected and analyzed in this study (Petrified Forest National Park Catalog Number PEFO 40757) was 21 cm long, and oval in cross section, approximately 29 × 23 cm (the slightly oval cross section is probably due to crushing during the burial and fossilization process); it weighed 31 kg (Fig. 1B). This section was one of five sections of a fossil stump. The two basal sections preserve portions of the root system. The next two distal sections above the base have fossil healing curls on both sides of the scar face; the top section preserves the top of the scar, with characteristic “cat face” arches similar to those often seen in modern fire scars (Fig. 1C, see Fig. S1). The total length of the scar from the base and what would have been the ground surface to the top is approximately 85 cm.

### Nearby trees with fire scar patterns

**Figure 2:**
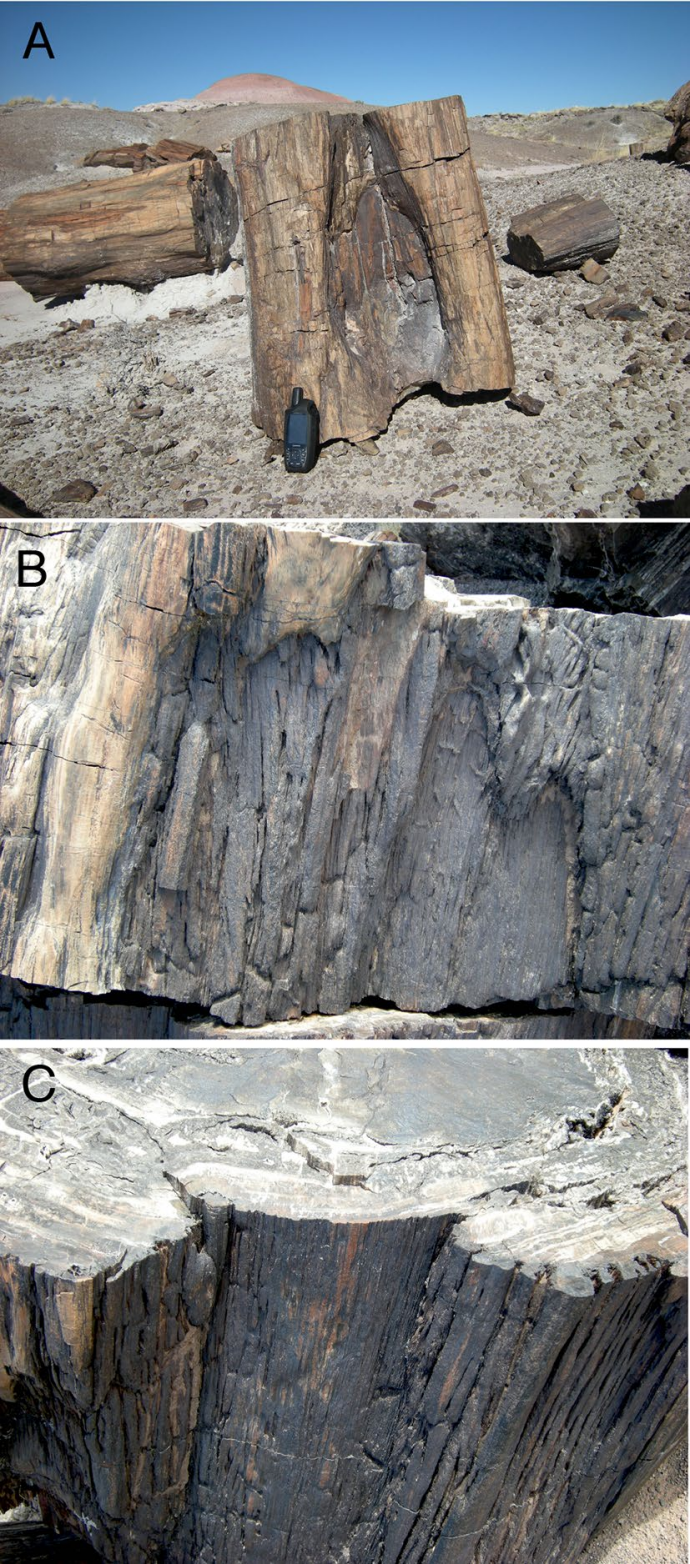
Fire-scar patterns on nearby fossil trees. (**A**) Basal cavity fire scar pattern (with 15 cm tall GPS unit for scale). (**B**) “Cat face” arches at top of another scar. (**C**) Woundwood overgrowth healing curls in trunk section below Fig. 2B section.

During our searches of the main fossil areas in Petrified Forest National Park in October 2013 and April 2014, we found 13 examples of fossil logs with external features resembling the healing curls and dry faces of modern fire-scarred trees among the several hundred fossil logs we observed. Three of those examples, including the specimen described here, were found in the Black Forest Bed. Because of the type of mineralization and preservation in that bed, the trees showed features of external morphology matching those of modern fire scars more clearly than examples from other areas of the park. All of the Black Forest examples were located at the base of the trunk, as in modern fire scars, and two of the three (Figs. 1C, 2B) showed the characteristic “cat face” arches at the top of the scar. Such features are common on modern fire-scarred trees and are explained by the flame dynamics of understory fires that scar trees without killing them^19^. A fossil trunk segment found approximately 20 m from the specimen described here had an arched cavity at the base of the tree that resembled modern fire scars in which a second fire has burned into the wood exposed in a previous fire scar (Fig. 2A). Another example located about 500 m away was on the basal section of a large tree approximately one meter in diameter. Arched patterns were visible on the top section (Fig. 2B) and distinct woundwood overgrowth lobes were visible below the top of the fossil scar (Fig. 2C).

### Scar-associated band

**Figure 3:**
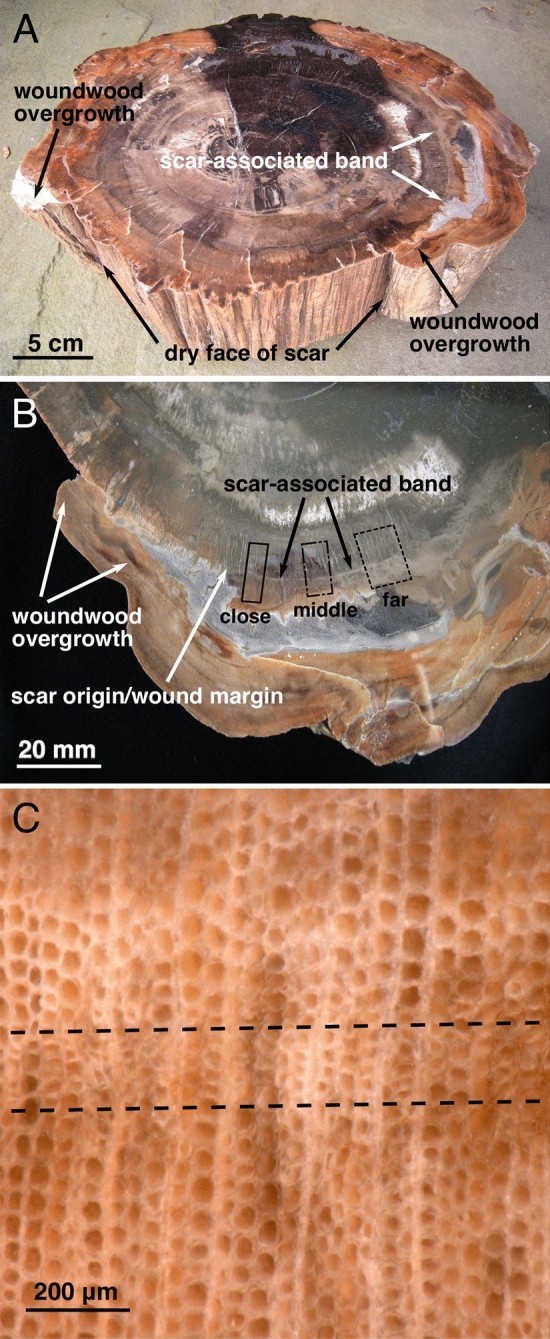
Full and magnified cross-sections of specimen. (**A**) Cross-section showing lobes of woundwood overgrowth over dry face of scar and scar-associated band. (**B**) Partial cross-section showing lobe of woundwood overgrowth, approximate scar origin, and visible scar-associated band at same radius as scar face; boxes indicate tangential regions where photomicrographs were made; note that transects in “middle” region were not visible beyond position 60 because a change in the mineralization of fossil wood in that area. (**C**) Photomicrograph showing area of tracheid distortion and compression that creates the visible of scar-associated ban.

The fossil wood we examined is homozylous and does not have visible growth rings; this is typical of *Agathoxylon arizonicum*, the dominant wood in Petrified Forest National Park^49^. A distinct, light-colored band was clearly visible in the fossil wood at the radius of the scarring event (Fig. 3A,B). Magnification showed that this band consisted of small and distorted tracheids (Fig. 3C), similar to the response to fire damage found in modern *Pinus ponderosa*^20^, and also observed in the first reported fire scar in the fossil record, from Bears Ears, Utah, another Late Triassic site in the Chinle Formation^21^. Heating of the trunk, as would occur in a surface fire, has been reported to cause permanent xylem deformation (tracheid compression and collapse) in modern trees^50^. Similar rings consisting of small and distorted tracheids associated with wounding have also been reported in early Permian fossil trees from southeastern Germany, although those could not be clearly linked with a specific cause of damage such as fire^51^.

### Pre-scarring drought signal in tracheid anatomy

**Figure 4:**
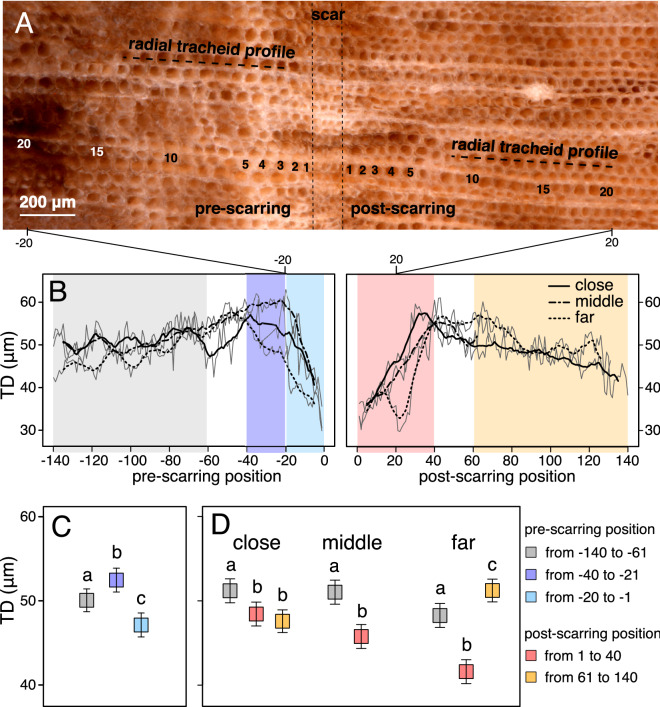
Magnified tracheids at scar-associated band, tracheid measurements and statistical analysis. (**A**) Photomicrograph showing 20 pre- and post-scarring tracheids; radial tracheid profiles and relative positions from the scar are shown. (**B**) Tracheid diameter (TD) displayed for each tracheid position related to scar-associated band (edge of band = position “0”) for pre-scarring and post-scarring sides for close, middle and far region from the woundwood overgrowth/scar margin. Data represented the mean (grey line) and 5-position moving average for “close” (solid line), “middle” (dashed-dotted line) and “far” (dashed line) region (see Fig. 3B). Background shading represents different regions coded with colors shown in the legend. Statistical comparison (**C**) to test pre-fire tracheid responses between average/normal pre-fire (positions − 140 to − 61), early pre-fire (positions − 40 to − 21) and immediate pre-fire conditions (positions − 20 to − 1), and (**D**) to test post-fire tracheid responses between average/normal pre-fire, immediate post-fire (positions 1 to 40), and later post-fire (positions 61 to 140) conditions at close, middle and far tangential regions. Data represents Least-square means (squares) ± SE (whiskers). Different letters indicate differences between periods according to Type III test of the linear mixed models in Tables S1, and S3 (*P* < 0.05).

From photomicrographs made along nine transects across the scar-associated band, we measured tracheid diameter, lumen diameter, and cell wall thickness in the pre-scarring and post-scarring fossil wood (Fig. 4A,B; see Methods and Supplementary Information for details). We observed a decrease (6–12%) in tracheid diameter and lumen diameter in the tracheids formed immediately before the scarring event (in positions ranging from -20 to -1 row prior to the scar-associated band), compared to tracheids that were formed earlier (in positions − 40 to − 21) or in normal pre-scarring conditions (positions − 140 to − 61; Fig. 4C and Tables S1, S2).

### Immediate post-fire decrease in tracheid size and subsequent increase following recovery

A 5–18% reduction in tracheid size (lumen and tracheid diameter) is present in the immediate post-scarring wood (in positions 1 to 40) compared to the average during pre-scarring conditions (positions − 140 to − 61), independent of the region analyzed (Fig. 4D and Tables S3, S4).

Analysis of tracheid measurements in our specimen showed larger tracheid (6%) and lumen (7%) diameters only in the “far” tangential region of the fossil wood after a recovery period (Fig. 4D and Tables S3, S4).

## Discussion

The specimen we describe has the distinctive external features of a fire scar. Internal wood anatomy of this specimen also shows features found in modern fire scars. A light-colored band composed of compressed and distorted tracheids was present in the fossil wood at the radius of the scarring event, as often seen in modern trees wounded by fire. Cell lumen diameter and cell wall thickness in the pre-scarring fossilized wood show a response similar to that described in modern conifers experiencing water stress and drought conditions^35–37^—a signal of drought conditions prevailing before the fire that scarred the ancient tree. Tracheids in the post-scarring wood exhibit an initial period of decreased size, similar to that reported for modern North American conifers, *Pinus ponderosa*, *Larix occidentalis*, and *Pseudotsuga menziesii* after wounding by fire^22^. Following this initial post-fire growth deficit, a growth increase indicated by larger tracheids was found in the fossil wood of this Petrified Forest specimen, as was the case in another Late Triassic fire scar from Bears Ears, Utah^21^. This is consistent with results from modern conifers^22,24^; a significant increase in tracheid size starting around two years after fire damage was reported in *Larix occidentalis* and *Pseudotsuga menziessii*^22^, the magnitude of which depended on the tangential position of the growing wood in relation to the wound margin (the farther from the margin, ranging from 0 to 4 cm in that study, the larger the effect).

The drought signal in the fire-scarred fossil wood we describe indicates climate variability on a short time scale (probably years); that low-intensity fires correlated with drought occurred in this area of the ancient Black Forest; and that some trees survived those fires. Two other fossil trees within a 500-m radius of the described specimen also show fire-scar features typically associated with low-severity fire regimes in modern forests^12,13,17,18^, suggesting this Late Triassic forest could have experienced such a fire regime. Recent research has shown a large-scale, long-term trend toward increasing temperature, aridity, and fires at around the time the fire-scarred tree we describe was growing^7,8^. Although a low-severity fire regime could occur in such a context, there is no clear or necessary connection between our findings and the evidence for those broader, longer-term trends. Although fire scars could be formed in a hot, dry climate, they would also be created in low-severity fire regimes in more temperate and moist climates.

Our results mean that the conditions favoring the evolution of traits adaptive in low-intensity surface fire regimes would have existed in the ancient Black Forest circa 210 Ma. Direct physical evidence for fire-adapted traits in Triassic fossils, such as thick bark, self-pruning growth form, epicormic sprouting, or serotiny, is scarce and inconclusive. In fact, fossil evidence of fire adaptation is scarce before the Early Cretaceous^11,14,57^, and it has been suggested that some fire-adapted traits are unlikely to produce direct fossil evidence^58^. Given the paucity of direct evidence of fire-adapted traits in the fossil record, the mapping of fire-adapted traits on dated molecular phylogenies is an emerging approach for understanding the timing and evolutionary origins of such traits. Such phylogenetic reconstructions suggest, for example, that thick bark originated in the genus *Pinus* around 126 Ma in association with low-intensity surface fires, and that alternative strategies of thick bark and branch shedding, or serotiny with branch retention, may have appeared around 89 Ma in response to more intense crown-fire regimes^57^. A more general phylogenetic reconstruction has been proposed to suggest that many early conifers were serotinous and that crown fire regimes may have dominated early conifer ecosystems from circa 350 Ma onwards because of the prevalence of highly flammable scale-leaved conifers^14^. Epicormic resprouting in eucalypts in Australia likely arose as an adaptation to fire approximately 60 Ma (ref.^59^). Fossil evidence of fire adaptations has corroborated phylogenetic predictions in the Proteoideae and the genus *Pinus*.

Very little evidence for fire adaptations in the Petrified Forest flora has been found to date. Two examples of fossil bark from the Black Forest Bed have been described. Both show bark with a scale-like appearance that somewhat resembles pine bark, up to 10 mm thick, on relatively small branches or trunks between 110 and 170 mm in diameter^49,52^. The distribution of branch bases on fossil trunks has been used to infer the probable growth form of the dominant conifer of the area, *Agathoxylon arizonicum*, but interpretations of this evidence differ. One study reported that well-preserved trunks of *Agathoxylon*, which were often 50 to 60 m tall, suggest that branches were usually restricted to the tops of the trees (as in some modern *Araucaria* species), suggesting they were self-pruning^53^. Another study concluded that lower branches might have carried viable foliage until the tree died^49^, but because fossilized foliage was lacking, there was no direct evidence on the matter. Although branch cores may have existed on the lower trunks of these fossil trees, it is impossible to know from the fossil evidence whether those branches were still alive when the tree reached its mature size or whether they were the bases of lower branches that were shed before the tree was fossilized. Fossil evidence of preventitious buds that could lead to epicormic sprouting—a common fire adaptation—has been found in *Woodworthia arizonica* from the Petrified Forest^54,55^. Fossil cones, characteristics of which might indicate serotiny, are quite rare in the Late Triassic Chinle Formation. A fossil ovulate cone has been described, with cone scales “covered with coaly residue indicating a thick and woody composition”^56^. This would not be inconsistent with serotiny, which has been proposed as an adaptation to a crown fire regime^14^, but it is not conclusive evidence either.

Whereas fossil evidence of fire adaptations has corroborated phylogenetic predictions in a few cases discussed in the previous paragraph, we are not aware of studies based on a deliberate search for direct fossil evidence of fire-adapted traits in the Triassic. A deliberate search for such fossil evidence could be productive and should be undertaken.

## Methods

### Search for fossil fire scars in Petrified Forest and discovery and preparation of specimen

In October, 2013, and April, 2014, we conducted a rapid reconnaissance in five major exposures of fossil logs in the Petrified Forest National Park, Arizona, under a National Park Service research permit. Among the hundreds of fossil log segments that we examined, we found 13 segments with external features similar to those of modern fire-scarred trees. They were distributed as follows: Black Forest N = 3; Blue Mesa N = 0; Rainbow Forest (Long Logs area) N = 4; Crystal Forest N = 4; Jasper Forest N = 2. All fossil trunks with possible fire scars were documented with photographs (see Supplementary Information, Figs. S2-S12), and GPS locations were recorded. This information is documented in research reports to Petrified Forest National Park and is available to qualified researchers with permission from the Superintendent. All work in the national park and with the specimen was conducted under permit # PEFO-2014-SCI-0002.

The specimen was removed from the Black Forest section of the park (Fig. 1). Initial examination of a polished cross section showed large areas with permineralized cellular structure and a visible scar-associated band in some areas. The large cross sections of the specimen were too large for a microscope stage, so a 2-cm cross section was further cut and polished (at the National Museum of Natural History, Smithsonian Institution, Washington, D.C.) to enable photomicroscopy of an area of woundwood overgrowth and with a section of the scar-associated band (Fig. 3). The specimen described here is housed in the collections of Petrified Forest National Park, Catalog Number PEFO 40757.

### Analysis of wood anatomy

We analyzed the wood anatomical features of the fossil specimen from photomicrographs made along nine 22 mm-long transects that crossed the scar-associated band at right angles, thus covering both pre- and post-scarring wood. Three transects of photomicrographs were made in each of three tangential regions along the scar-associated band at progressively greater distances from the scar origin; the “close” region was centered approximately one centimeter from the wound margin/scar origin, the “middle” region center at three cm, and the “far” region at approximately five cm (see Fig. 3B). A total of 12,410 tracheids were measured, calculating radial lumen diameter (LD), cell wall thickness (CWT) and radial tracheid diameter (TD) with the “tgram” R package (available from CRAN; https://cran.r-project.org;^33^). We also recorded the relative position of each tracheid from the edge of the scar-associated band in each direction, from the closest position 1 to the farthest position 140 (Fig. 4A,B). We fitted linear mixed models to test the hypotheses of the study based on differences in wood anatomical features (LD, CWT and TD) of tracheids before and after scarring. First, we tested the pre-fire drought hypothesis by comparing the pre-scarring tracheids in the relative positions − 20 to − 1 with − 140 to − 61 and − 40 to − 21 from the scar (see Fig. 4, Tables S1, S2). Second, we tested hypotheses about immediate post-fire growth reduction and subsequent growth increase by comparing tracheids in the pre-scarring -140 to -61 with post-scarring 1 to 40 and 61 to 140 positions from the scar at the three tangential regions (close, middle or far from the woundwood overgrowth/scar margin, see Fig. 4, Tables S3, S4). The anatomical features of the pre-scarring tracheids in the relative positions − 140 to − 61 were used as control for the normal xylem growth, and then, included in both models. Further details of methods are provided in the appendix of Supporting Information.

## Supplementary information

Supplementary Information.
