# Supplementary material for: Fire-scarred fossil tree from the Late Triassic shows a pre-fire drought signal
Source: Sci Rep. 2020 Nov 18;10:20104. doi: 10.1038/s41598-020-77018-w (PMC7676234; doi:10.1038/s41598-020-77018-w)
Supplement: Supplementary file 1 — Supplementary Information. [file 41598_2020_77018_MOESM1_ESM.docx]

**Fire-scarred fossil tree from the Late Triassic** **shows a pre-fire drought signal**

**Bruce A. Byers^1^*, Lucía DeSoto^2,3^, Dan Chaney^4^, Sidney R. Ash^5^, Anya B. Byers^6^, Jonathan B. Byers^7^, Markus Stoffel^8,9,10^**

**^1^ Bruce Byers Consulting, Falls Church, Virginia, USA**

**^2^ MedDendro Lab, Centre for Functional Ecology, University of Coimbra, Coimbra, Portugal**

**^3^ Experimental Station of Arid Zones, Spanish National Research Council (CSIC), Spain**

**^4^ National Museum of Natural History, Washington, DC, USA**

**^5^ Deceased February 2019; formerly University of New Mexico, Albuquerque, NM, USA**

**^6^ The Nature Conservancy, Boulder, CO, USA**

**^7^ Department of Geography, University of Montana, Missoula, MT, USA**

**^8^ Climate Change Impacts and Risks in the Anthropocene, Institute for Environmental Sciences, University of Geneva, Switzerland**

**^9^ dendrolab.ch, Department of Earth Sciences, University of Geneva, Switzerland**

**^10^ Department F.-A. Forel for Aquatic and Environmental Sciences, University of Geneva, Switzerland**

**Supplementary Information**

**Materials and Methods.**

**Search for Fossil Fire Scars.** We searched for examples of fossil logs with external features (healing curls, dry scar faces, “cat face” arches at top of scar) resembling modern fire-scarred trees (Fig. S1) both on and off trails in the main fossil areas of Petrified Forest National Park (Rainbow Forest/Long Logs, Crystal Forest, Jasper Forest, Blue Mesa, and Black Forest) in October 2013 and April 2014. We examined a total of several hundred fossil logs and found 13 examples of possible fossil fire scars. Three of those, from Black Forest, are pictured in the main text; the ten not pictured there are shown below (Figs. S2-S12). Locations of all examples are on file at the national park.

**
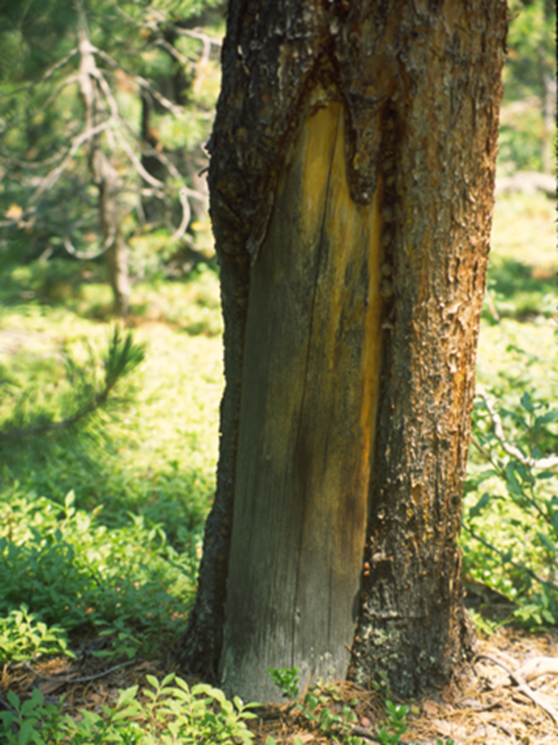
**

**Fig. S1. Fire scar on lodgepole pine (*Pinus contorta*), Boulder County, Colorado, USA.** Note healing curls of woundwood overgrowth on both sides of dry face of the scar, and “cat face” arches at top of scar.

**
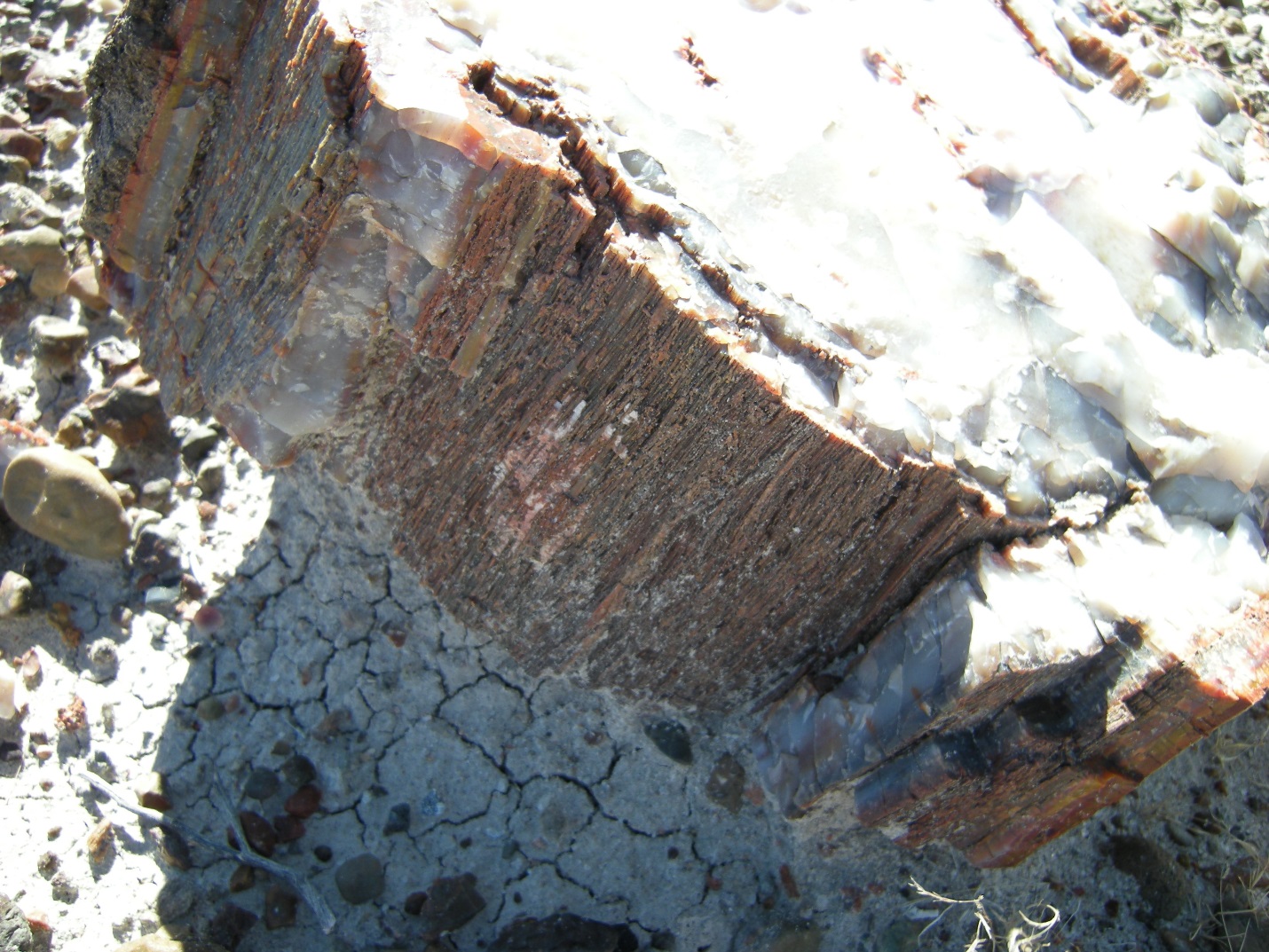
**

**dry face of scar**

**healing curl**

**scar**

**Fig. S2. Crystal Forest Example 1.**

**
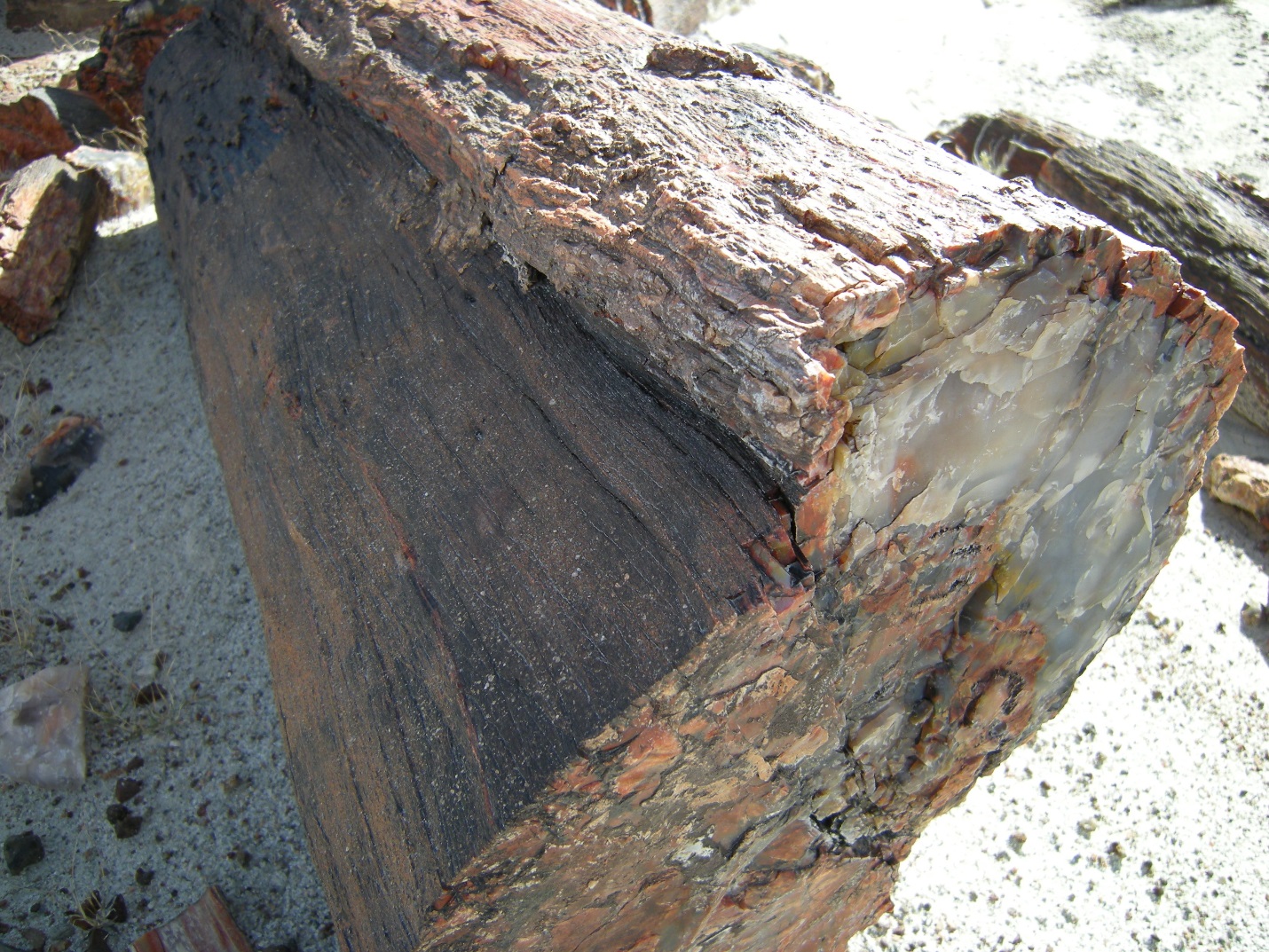
**

**dry face of scar**

**healing curl**

**Fig. S3. Crystal Forest Example 2.**

**
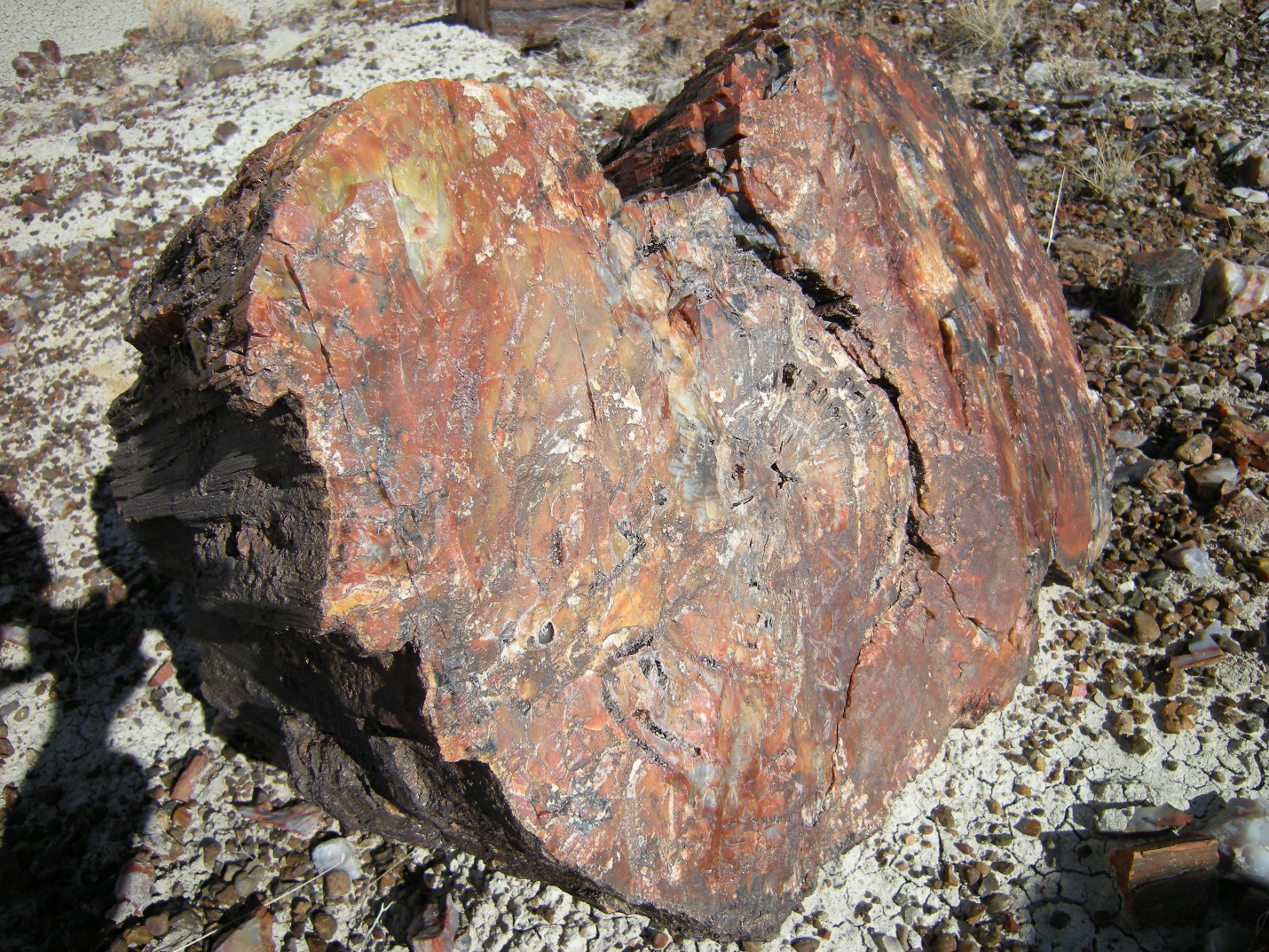
**

**healing curl**

**healing curl**

**dry face of scar**

**Fig. S4. Crystal Forest Example 3.**

**
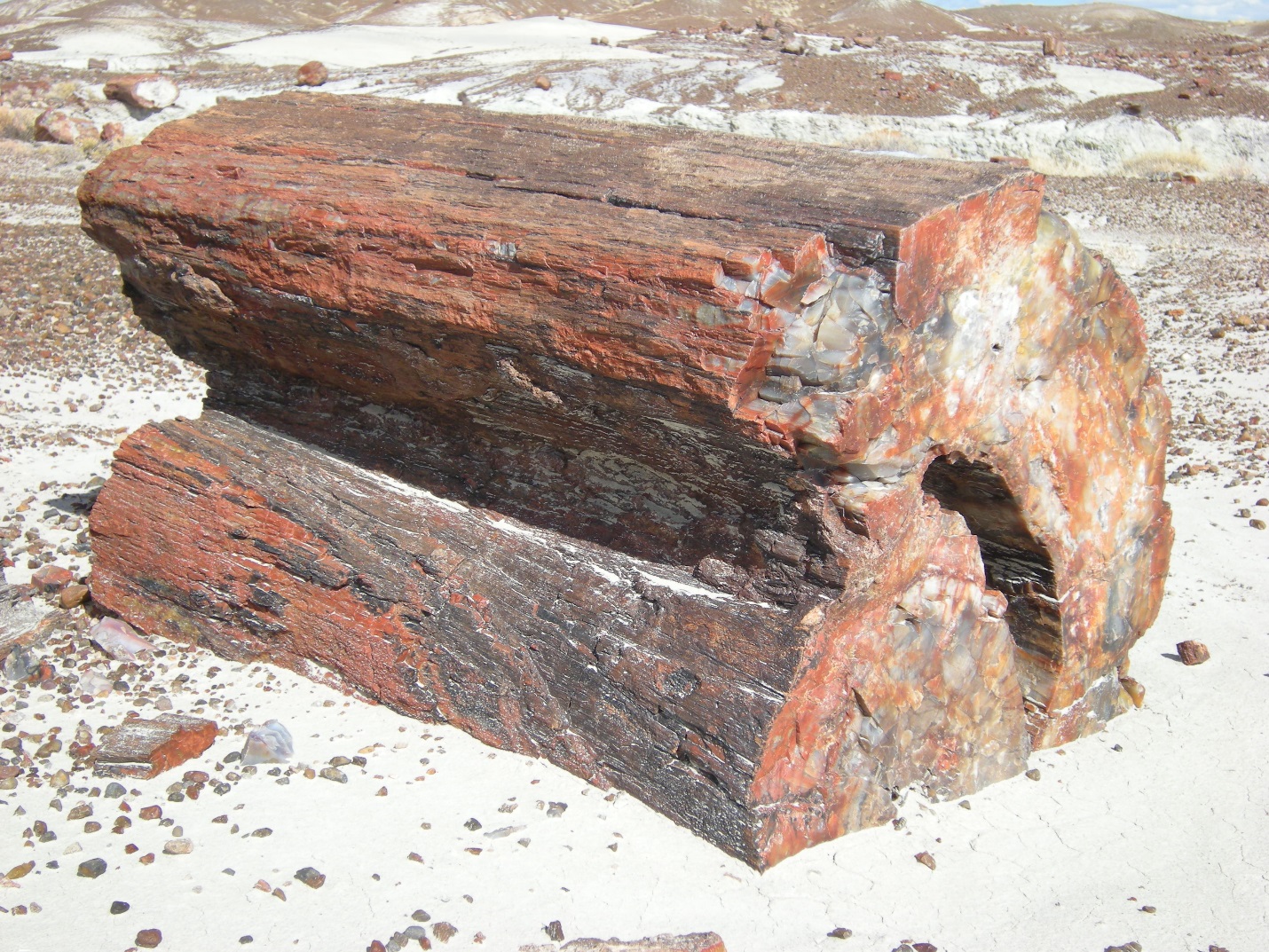
**

**dry face of scar**

**healing curl**

**healing curl**

**Fig. S5. Crystal Forest Example 4.**

**
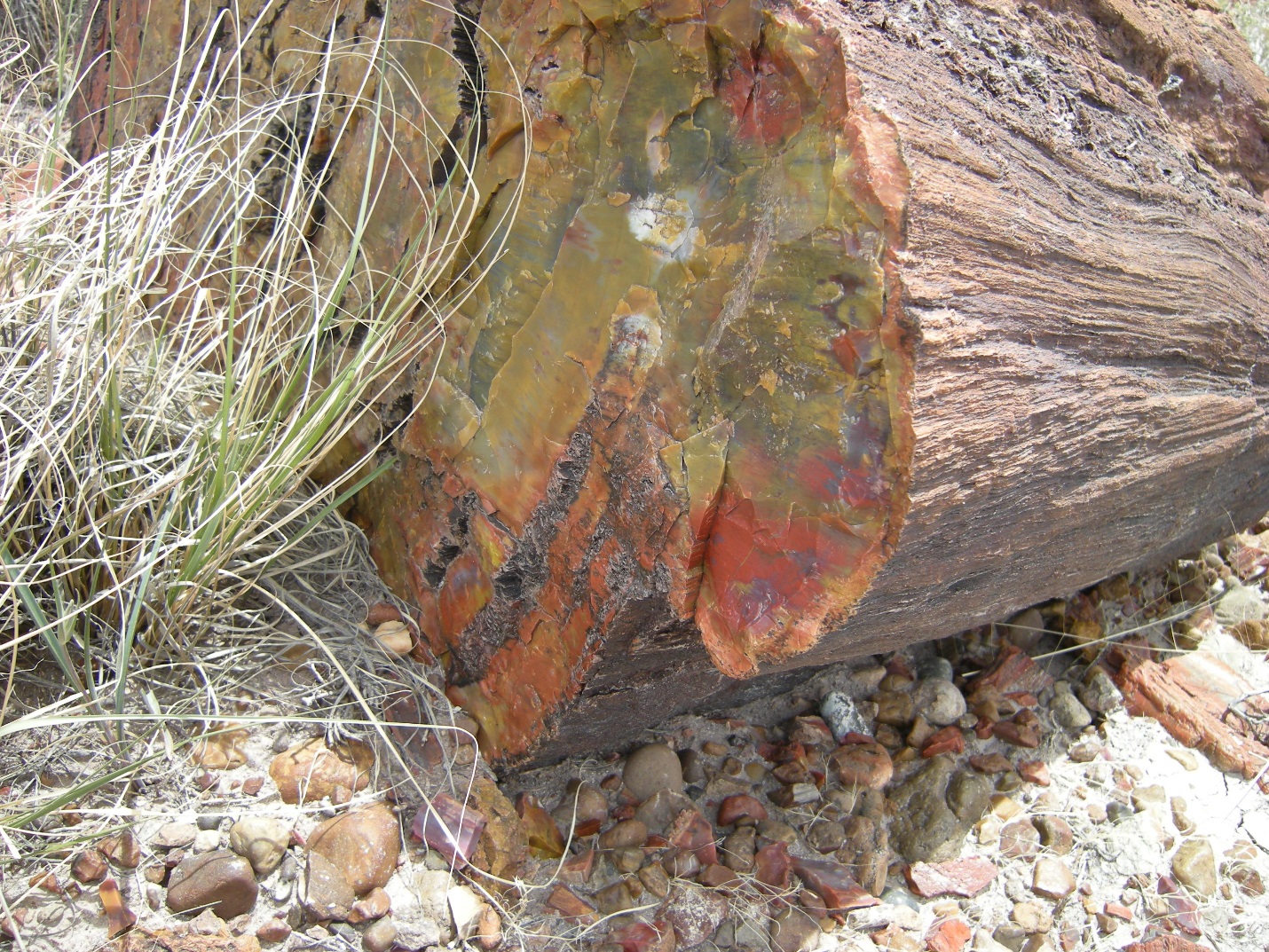
**

**healing curl**

**dry face of scar**

**Fig. S6. Jasper Forest Example 1.**

**
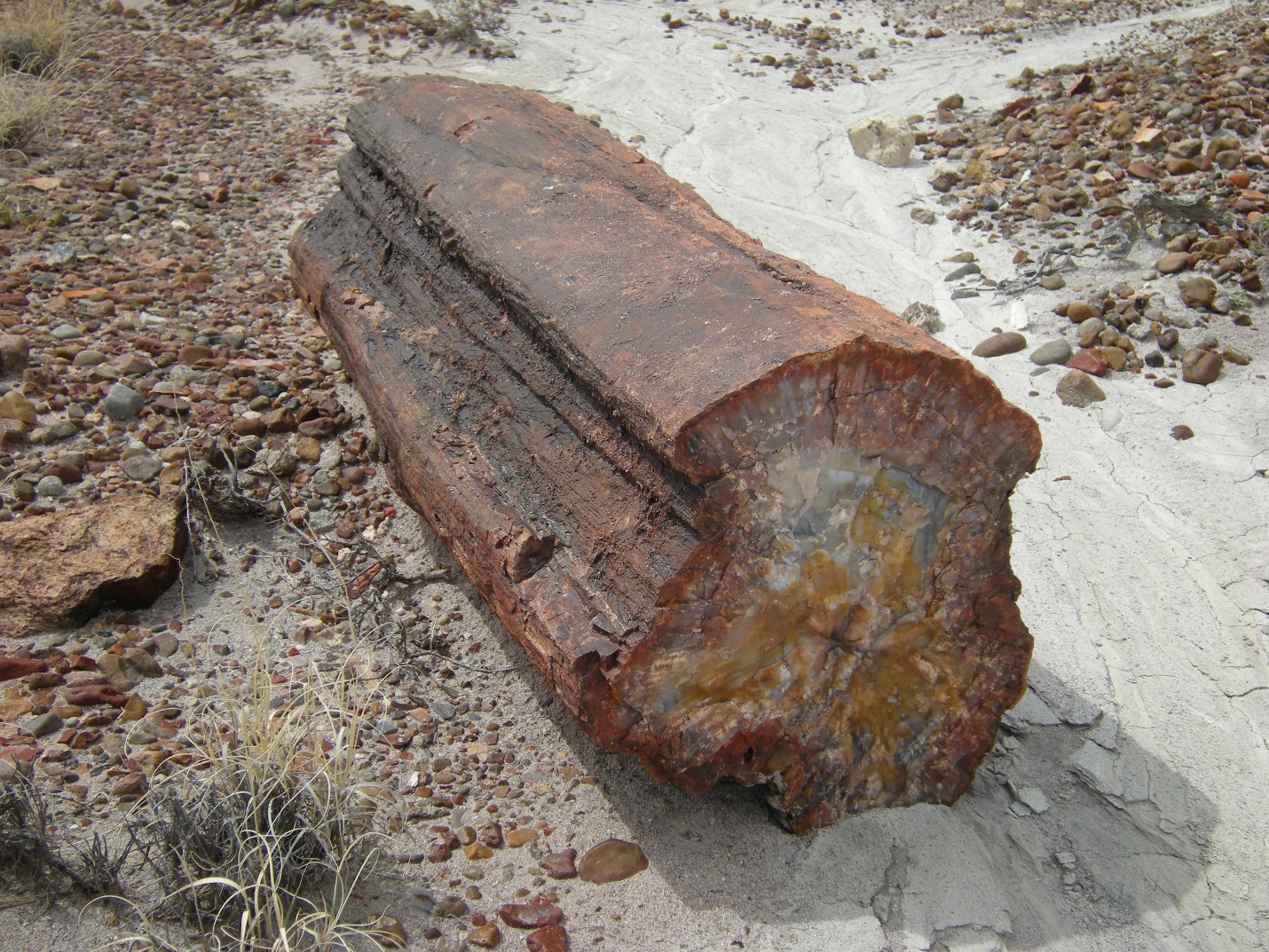
**

**dry face of scar**

**healing curl**

**healing curl**

**Fig. S7. Jasper Forest Example 2.**

**
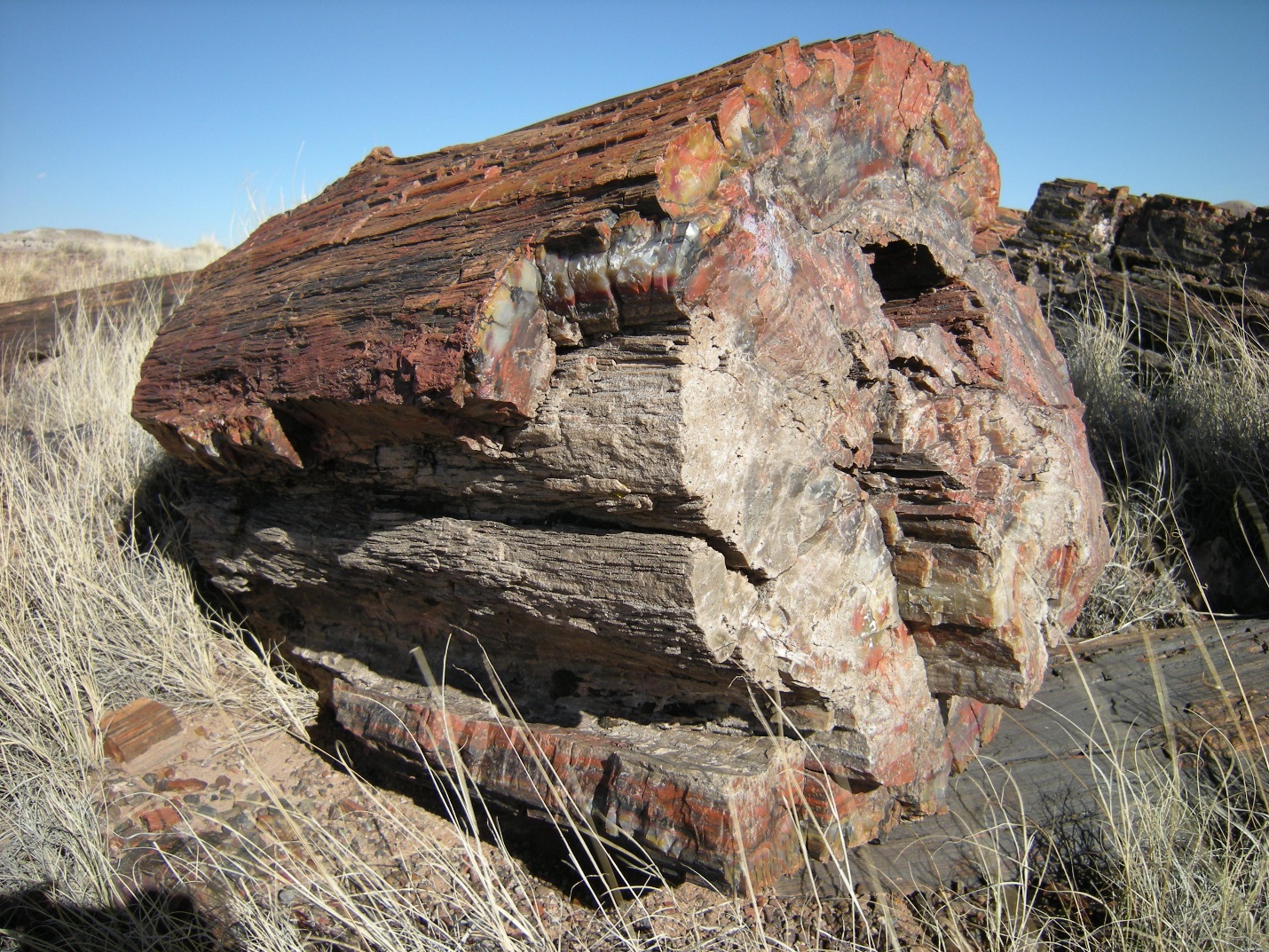
**

**healing curl**

**healing curl**

**dry face of scar**

**Fig. S8. Rainbow Forest/Long Logs Example 1.**

**
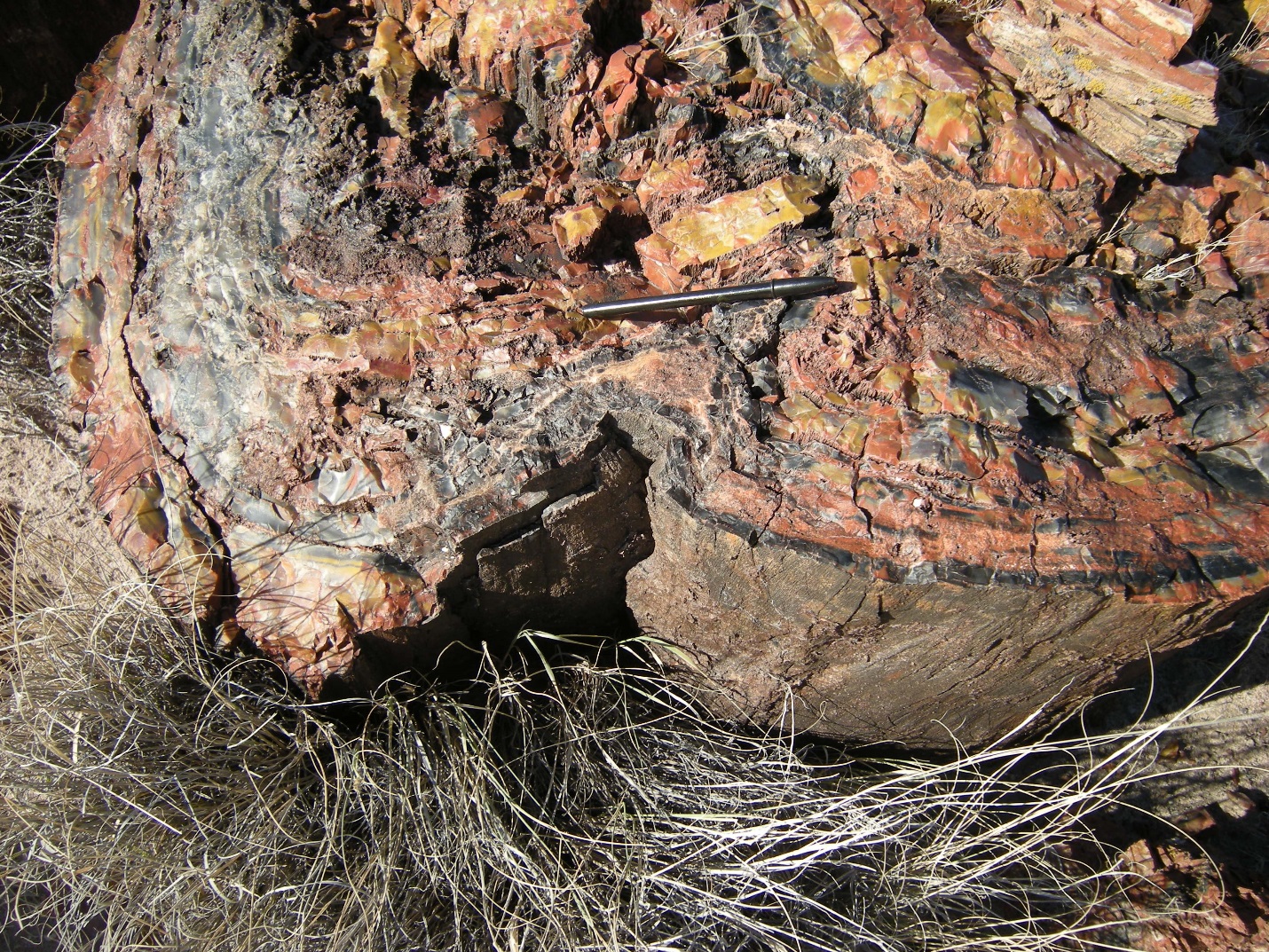
**

**dry face of scar**

**healing curl**

**healing curl**

**Fig. S9. Rainbow Forest/Long Logs Example 2.** Pen (15 cm) for scale.

**
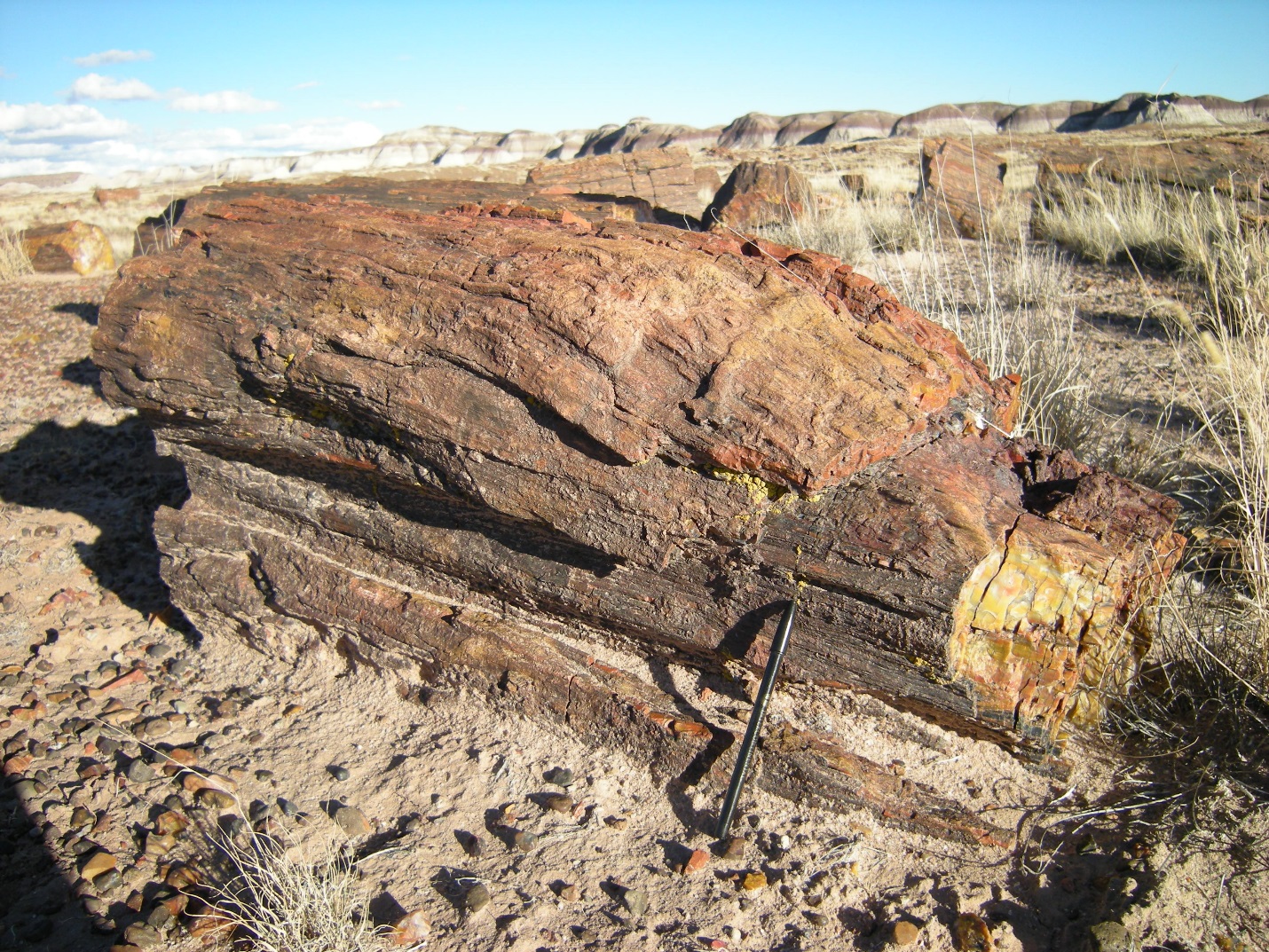
**

**healing curl**

**healing curl**

**dry face of scar**

**Fig. S10. Rainbow Forest/Long Logs Example 3, Side View.** Pen (15 cm) for scale.

**
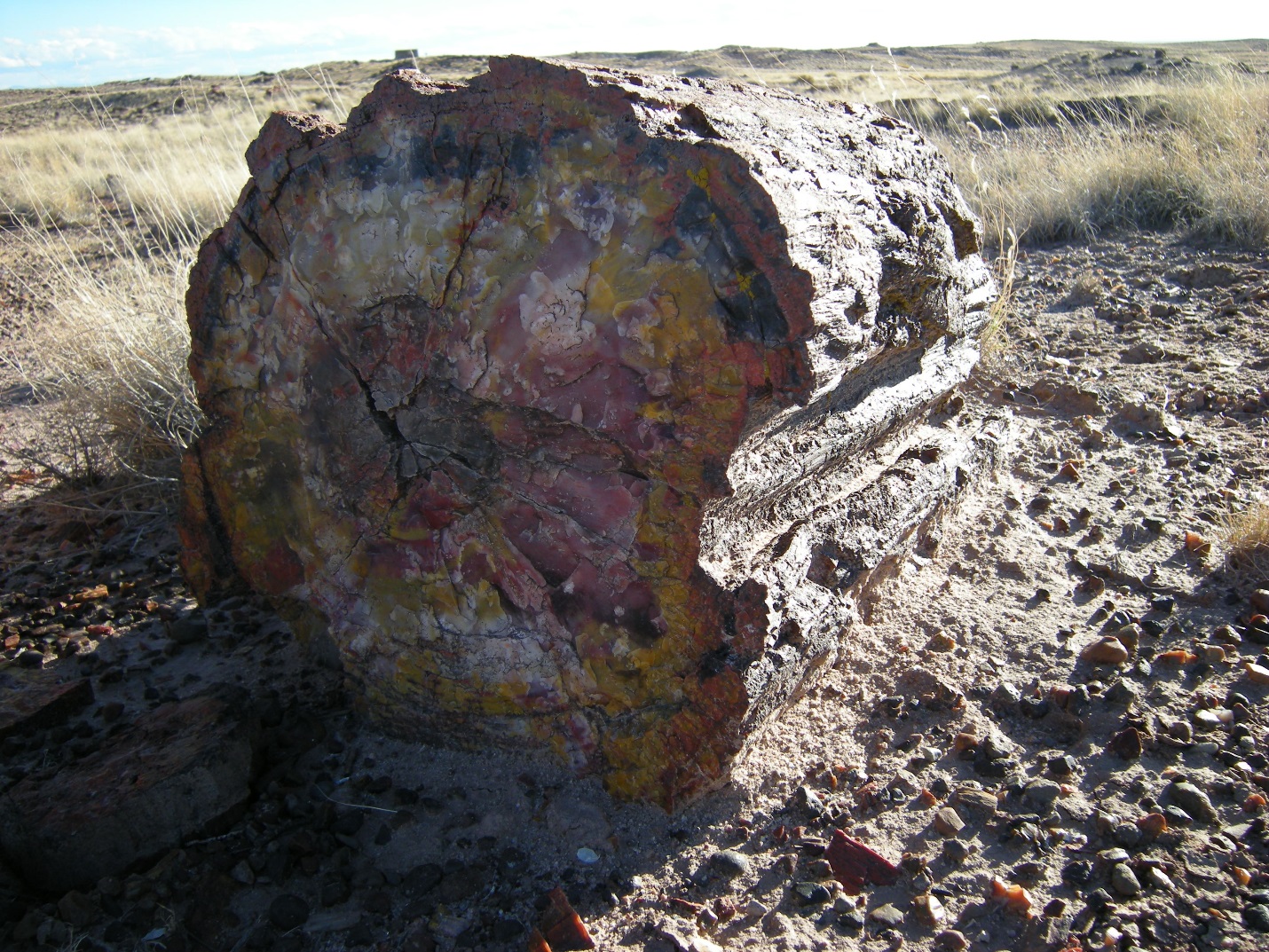
**

**healing curl**

**healing curl**

**dry face of scar**

**Fig. S11. Rainbow Forest/Long Logs Example 3, End View.**

**
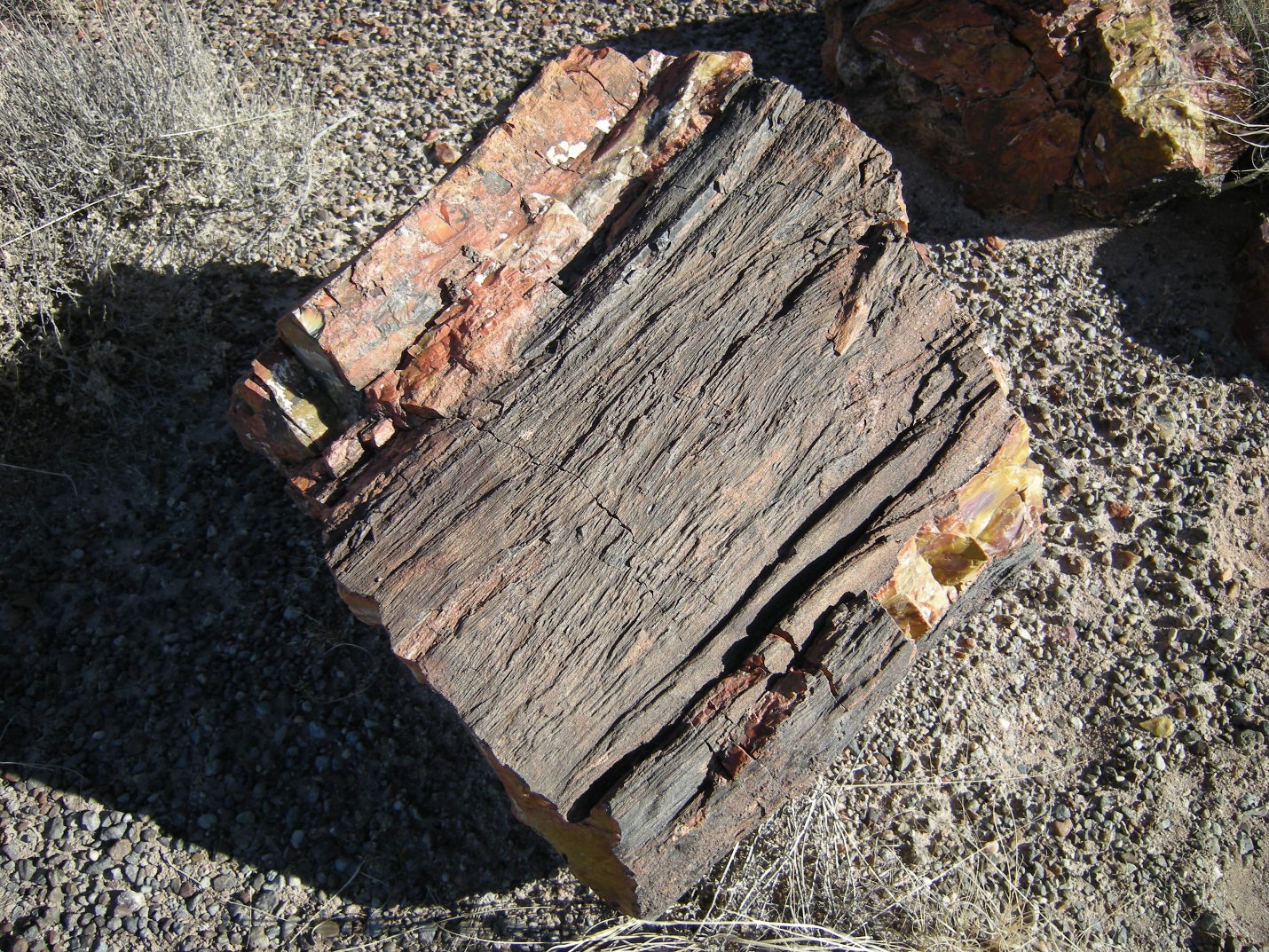
**

**healing curl**

**healing curl**

**dry face of scar**

**Fig S12. Rainbow Forest/Long Logs Example 4.**

**Analysis of Wood Anatomy.** We analyzed the wood anatomical features of the fossil specimen from photomicrographs made along nine 22mm-length transects that crossed the scar-associated band at right angles, thus covering both pre- and post-scarring wood. Three transects were made in each of three tangential regions along the scar-associated band at progressively greater distances from the scar origin; the “close” region was centered approximately one centimeter from the wound margin/scar origin, the “middle” region center at three cm, and the “far” region at approximately five cm (see Fig. 5) One-hundred-ninety-eight (198) 1x1mm photomicrographs (22 per transect; RGB, color 24 bit) were taken using a Olympus SZX-12 MDU motorized stereozoom microscope with 1.2x lens and recorded using an Olympus Q-Color 5Mp camera with an image scale of 0.3 μm/pixel. We plotted 868 radial tracheid profiles, 587 pre- and 321 post-scarring, using the image analysis program Image J (version 1.48, Abramoff et al. 2004 Image processing with ImageJ. Biophoton. Int. **11**, 36–42, Fig. 2). Tracheid cell walls and lumen diameters were discriminated based on grey values along a radial line passing through the tracheid profiles (DeSoto et al. 2011 Intra-annual patterns of tracheid size in the Mediterranean tree *Juniperus thurifera* as an indicator of seasonal water stress. Can J For Res **41**,1280–1294.). To attain uniform measurements, only the tangentially largest cells in clearly-focused radial tracheid files were measured, and unfocused or deformed cells were not included. The radial lumen diameter (LD), cell wall thickness (CWT) and radial tracheid diameter (TD) were calculated with the “tgram” R package, (available from CRAN; http://cran.r-project.org; DeSoto et al. 2011). A total of 12,410 tracheids were measured. For every radial tracheid file, we recorded the relative position of each tracheid from the edge of the scar-associated band in each direction, from the closest position (1) to the farthest position (140).

Two Linear Mixed Models (LMMs, Quinn and Keough 2002 Experimental design and data analysis for biologists. Cambridge University Press, Cambridge, UK) were used to test the hypotheses of the study based on differences in wood anatomical features before and after scarring. First, we tested the pre-fire drought hypothesis by comparing the anatomical features of the pre-scarring tracheids in the relative positions -140 to -61, -40 to -21 and -20 to -1 from the scar. We included scarring relative position as fixed effect, with the tracheid position nested in tracheid radial profile nested in transect nested in region as random effect. Second, we tested immediate post-fire growth reduction and subsequent growth increase hypotheses by comparing the anatomical features of the tracheids in the pre-scarring positions (-140 to -61) and post-scarring positions (1 to 40 and 61 to 140) relative to the scar in three tangential regions (close, middle or far from the woundwood overgrowth/scar margin). We included region and position relative to the scar and their interactions as fixed effects, with the tracheid position nested in tracheid radial profile nested in transect as random effect. Region was considered as fixed effect in the latter models, rather than as a random effect (as in the former models), because we wanted to test the differences between them. We assumed a normal error distribution with an identity link function for LD, TD and CWT in all models. The anatomical features of the pre-scarring tracheids in relative positions -140 to -61 were used as a control for the normal xylem growth, and then included in all models. We fitted LMMs using the “lme4” and “car” R packages (available from CRAN; Bates et al. 2015 Fitting Linear Mixed-Effects Models Using *lme4*. J. Stat. Softw. 67, 51; Fox & Weisberg 2011 An R companion to applied regression. Second. Sage, Thousand Oaks CA, USA). Least-square means were obtained for pre- and post-scarring sides, and the differences between them were tested pairwise using the “lsmeans” R package (available from CRAN; Lenth 2016 Least-Squares means: The R Package *lsmeans*. J. Stat. Softw. **69**, 1–33).

**Table S1.** **Testing Pre-fire Water Stress/Drought Hypothesis.** Results of Type III fixed effects test of the mixed linear model for tracheid diameter (TD), lumen diameter (LD) and cell wall thickness (CWT) of the tracheids located in three sections of pre-scarring positions ranged -140 to -61, -40 to -21 and -20 to -1 relative positions from the scar. Data represent the degrees of freedom (df num, df den), the F-statistic with the associated P-value of significance (significant effects, P < 0.05; total number of tracheids = 5,922) for factor scarring position, and random factor position nested in radial tracheid profile nested in transect nested in region (close, middle, and far).

|  | **TD** | | | |  | **LD** | | | |  | **CWT** | | | |
| --- | --- | --- | --- | --- | --- | --- | --- | --- | --- | --- | --- | --- | --- | --- |
|  | df | df | *F* | *P* |  | df | df | *F* | *P* |  | df | df | *F* | *P* |
| **Fixed effects** | num | dem |  |  |  | num | dem |  |  |  | num | dem |  |  |
| scarring position | 2 | 997.7 | 39.47 | < 0.001 |  | 2 | 978.6 | 40.1 | < 0.001 |  | 2 | 1001.3 | 14.66 | < 0.001 |
|  |  |  |  |  |  |  |  |  |  |  |  |  |  |  |
| **Random effects** |  |  | *Chi*2 | *P* |  |  |  | *Chi*2 | *P* |  |  |  | *Chi*2 | *P* |
| region (transect (profile (position))) | | 2 | 68.28 | < 0.001 |  |  | 2 | 69.18 | < 0.001 |  |  | 2 | 27.21 | < 0.001 |
|  |  |  |  |  |  |  |  |  |  |  |  |  |  |  |

**Table S2. Testing Pre-fire Water Stress/Drought Hypothesis.** Least-square means and confidence intervals (C.I.) of wood anatomical features, radial tracheid diameter (TD), lumen diameter (LD), and cell wall thickness (CWT) of the pre-scarring positions. Statistical differences (P < 0.05) base on the generalized linear model in Table S1 are shown with different letters.

| **feature** | **scarring position** | **Ls-mean** | **C.I.** |  |
| --- | --- | --- | --- | --- |
| **TD** | -61 – -140 | 50.06 | 45.46 - 54.66 | a |
|  | -21 – -40 | 52.46 | 48.17 - 56.76 | b |
|  | -1 – -20 | 47.13 | 42.85 - 51.41 | c |
| **LD** | -61 – -140 | 34.35 | 31.57 - 37.13 | a |
|  | -21 – -40 | 36.8 | 34.28 - 39.32 | b |
|  | -1 – -20 | 32.29 | 29.74 - 34.76 | c |
| **CWT** | -61 – -140 | 7.84 | 6.72 - 8.96 | a |
|  | -21 – -40 | 7.86 | 6.78 - 8.94 | a |
|  | -1 – -20 | 7.35 | 6.28 - 8.42 | b |

**Table S3.** **Testing Post-scarring Immediate Growth Reduction and Later Growth Increase Hypotheses.** Results of Type III fixed effects test of the mixed linear model for tracheid diameter (TD), lumen diameter (LD) and cell wall thickness (CWT) of the tracheids located in three sections of pre-scarring positions ranged -141 – -61, and of post-scarring positions ranged 1 – 40 and 61 – 140 relative to the scar. Data represent the degrees of freedom (df num, df den), the F-statistic with the associated P-value of significance (significant effects, P < 0.05; total number of tracheids = 8,055) for factors region (close, middle, and far), scarring position, and its interaction, and random effect position nested in radial tracheid profile nested in transect.

|  | **TD** | | | |  | **LD** | | | |  | **CWT** | | | |
| --- | --- | --- | --- | --- | --- | --- | --- | --- | --- | --- | --- | --- | --- | --- |
|  | df | df | *F* | *P* |  | df | df | *F* | *P* |  | df | df | *F* | *P* |
| Fixed effects | num | dem |  |  |  | num | dem |  |  |  | num | dem |  |  |
| region | 2 | 11.6 | 3.03 | 0.087 |  | 2 | 12.4 | 2.79 | 0.1 |  | 1 | 10 | 2.28 | 0.153 |
| scarring position | 2 | 617.6 | 6.9 | 0.001 |  | 2 | 617.1 | 7.58 | < 0.001 |  | 1 | 617 | 6.43 | 0.002 |
| region × scarring position | 3 | 606.2 | 13.67 | < 0.001 |  | 3 | 601.2 | 12.15 | < 0.002 |  | 1 | 608.9 | 7.46 | < 0.001 |
|  |  |  |  |  |  |  |  |  |  |  |  |  |  |  |
| Random effects |  |  | *Chi*2 | *P* |  |  |  | *Chi*2 | *P* |  |  |  | *Chi*2 | *P* |
| transect (profile (position)) |  | 7 | 107.87 | < 0.001 |  |  | 7 | 121.42 | < 0.001 |  |  | 7 | 32.3 | < 0.001 |

**Table S4.** **Testing Post-scarring Immediate Growth Suppression and Later Growth Release Hypotheses.** Least-square means and confidence intervals (C.I.) of wood anatomical features, radial tracheid diameter (TD), lumen diameter (LD), and cell wall thickness (CWT) of scarring position periods (average/normal pre-fire conditions, positions -140 to -61; early pre-fire conditions, positions -40 to -21; immediate pre-fire conditions, positions -20 to -1; immediate post-fire period, positions 1 to 40; later post-fire period, positions 61 to 140) at close, middle and far regions. Statistical differences (P < 0.05) between periods based on the generalized linear model in Table S3 are shown with different letters for each region.

| **feature** | **region** | **scarring position** | **Ls-mean** | **C.I.** |  |
| --- | --- | --- | --- | --- | --- |
| **TD** | **close** | -61 – -140 | 51.19 | 49.02-53.36 | a |
|  |  | 1 – 40 | 48.43 | 45.93-50.93 | b |
|  |  | 61 – 140 | 47.58 | 45.32-49.83 | b |
|  | **middle** | -61 – -140 | 51.03 | 48.91-53.14 | a |
|  |  | 1 – 40 | 45.76 | 43.47-48.04 | b |
|  |  | 61 – 140 | -- | -- |  |
|  | **far** | -61 – -140 | 48.25 | 46.18-50.32 | a |
|  |  | 1 – 40 | 41.58 | 39.21-43.95 | b |
|  |  | 61 – 140 | 51.22 | 48.88-53.57 | c |
| **LD** | **close** | -61 – -140 | 35.55 | 33.99-37.11 | a |
|  |  | 1 – 40 | 32.36 | 30.49-34.22 | b |
|  |  | 61 – 140 | 33.05 | 31.41-34.69 | b |
|  | **middle** | -61 – -140 | 34.5 | 32.98-36.01 | a |
|  |  | 1 – 40 | 30.51 | 28.83-32.18 | b |
|  |  | 61 – 140 | -- | -- |  |
|  | **far** | -61 – -140 | 33.23 | 31.76-34.71 | a |
|  |  | 1 – 40 | 27.12 | 25.38-28.87 | b |
|  |  | 61 – 140 | 35.64 | 33.91-37.36 | c |
| **CWT** | **close** | -61 – -140 | 7.82 | 7.25-8.38 | a |
|  |  | 1 – 40 | 8.04 | 7.43-8.65 | a |
|  |  | 61 – 140 | 7.26 | 6.68-7.84 | b |
|  | **middle** | -61 – -140 | 8.26 | 7.7-8.82 | a |
|  |  | 1 – 40 | 7.62 | 7.03-8.2 | b |
|  |  | 61 – 140 | -- | -- |  |
|  | **far** | -61 – -140 | 7.51 | 6.96-8.07 | ab |
|  |  | 1 – 40 | 7.21 | 6.62-7.8 | a |
|  |  | 61 – 140 | 7.77 | 7.18-8.36 | b |
